# Impact of body mass index in patients with tricuspid regurgitation after transcatheter edge-to-edge repair

**DOI:** 10.1007/s00392-023-02312-2

**Published:** 2023-10-04

**Authors:** Johanna Vogelhuber, Tetsu Tenaka, Mitsumasa Sudo, Atsushi Sugiura, Can Öztürk, Refik Kavsur, Anika Donner, Georg Nickenig, Sebastian Zimmer, Marcel Weber, Nihal Wilde

**Affiliations:** Heart Center Bonn, Department of Medicine II, University Hospital Bonn, Venusberg-Campus 1, 53127 Bonn, Germany

**Keywords:** Tricuspid valve disease, Tricuspid regurgitation, Transcatheter edge-to-edge repair, Body mass index

## Abstract

**Background:**

Obesity and underweight represent classical risk factors for outcome in patients treated for cardiovascular disease. This study describes the impact of different body mass index (BMI) categories on 1-year clinical outcome in patients with tricuspid regurgitation (TR) undergoing transcatheter-edge-to-edge repair (TEER).

**Methods:**

We analyzed 211 consecutive patients (age 78.3 ± 7.2 years, 55.5% female, median EuroSCORE II 9.6 ± 6.7) with tricuspid regurgitation undergoing TEER from June 2015 until May 2021. Patients were prospectively enrolled in our single center registry and were retrospectively analyzed. Patients were stratified according to body mass index (BMI) into 4 groups: BMI < 20 kg/m^2^ (underweight), BMI 20.0 to < 25.0 kg/m^2^ (normal weight), BMI 25.0 to > 30.0 kg/m^2^ (overweight) and BMI ≥ 30 kg/m^2^ (obese).

**Results:**

Kaplan–Meier survival curves demonstrated inferior survival for underweight and obese patients, but comparable outcomes for normal and overweight patients (global log rank test, *p* < 0.01). Cardiovascular death was significantly higher in underweight patients compared to the other groups (24.1% vs. 7.0% vs. 6.3% vs. 6.4%; *p* < 0.01). Over all, there were comparable rates of bleeding, stroke and myocardial infarction. Multivariable Cox regression analysis (adjusted for age, gender, coronary artery disease, chronic obstructive pulmonary disease, tricuspid annular plane systolic excursion, left-ventricular ejection fraction) confirmed underweight (HR 3.88; 95% CI 1.64–7.66; *p* < 0.01) and obesity (HR 3.24; 95% CI 1.37–9.16; *p* < 0.01) as independent risk factors for 1-year all-cause mortality.

**Conclusions:**

Compared to normal weight and overweight patients, obesity and underweight patients undergoing TEER display significant higher 1-year all-cause mortality.

**Graphical abstract:**

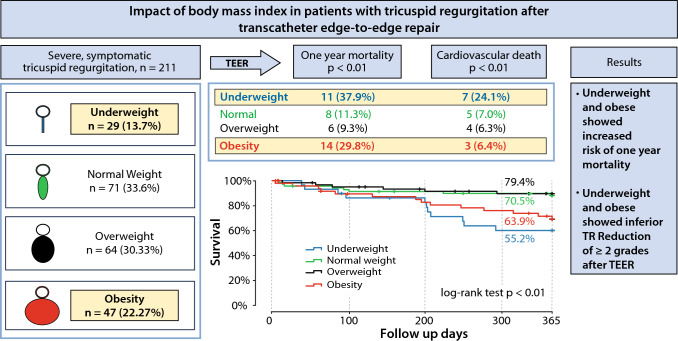

**Supplementary Information:**

The online version contains supplementary material available at 10.1007/s00392-023-02312-2.

## Introduction

Tricuspid regurgitation is a common finding in routine practice as population-based studies showed that the prevalence of tricuspid regurgitation of any grade amounts to > 80% of the population, particularly affecting people at older age and of female gender [1, 2]. Consequently, and with regard to the elderly (> 70 years), a significant TR (moderate) was present in 1.5% of male and 5.6% of female patients, respectively [2]. Thus, clinically relevant TR can be anticipated in approximately 3 Mill. individuals in Europe and 1.5 Mill. individuals in the USA [2–4]. Moreover, prevalence of 3 moderate TR in patients with chronic heart failure and reduced left ventricular ejection fraction is even higher with approximately 26% [2, 5, 6]. The importance of TR for prognosis has long been underrated and treatment has subsequently been neglected in accordance with the initial recommendations to handle TR with optimal heart failure therapy [4, 7, 8]. However, numerous studies recently underlined congruently the negative impact of significant TR on morbidity and mortality if left untreated [4, 6, 9–13].

Underweight and obesity are known risk factors for adverse outcome in patients with cardiovascular diseases [14, 15]. With regard to valvulopathies, recent studies showed an increased morbidity and mortality for the overweight and obese patient cohort after transcatheter aortic valve replacement (TAVR) as well as for underweight patients after transcatheter edge-to-edge repair (TEER) for mitral regurgitation, respectively [16, 17].

Concerning the prognostic impact of significant TR for the clinical course, identification and characterization of relevant risk constellations have more recently come to the fore—especially in the light of selecting the suitable treatment strategy for each patient. In terms of patient selection, established scores for left-sided valvulopathies and/or CABG procedures such as STS Score and the EUROScore II are not validated and imprecise concerning accurate risk stratification of tricuspid valve procedures although still recommended for perioperative risk assessment especially in the elderly and high-risk population [18]. Currently, the TRISCORE is most frequently used to assess procedural risk for tricuspid valve procedures as it includes and addresses parameters of right heart failure such as impaired liver function as a result of an increased central venous backlog, daily dosage of diuretics and right heart failure signs [19, 20]. However, parameters such as frailty which are associated with adverse outcome and BMI are not included into this score. While defining frailty still remains vague and without a predefined gold standard, BMI acquisition and interpretation is simple to attain and less faulty. But up to now, little is known regarding the impact of underweight and obesity on the postinterventional course after edge-to-edge repair for significant symptomatic TR.

## Methods

### Study population

This study was designed as a retrospective analysis of data from the Bonn registry, which is a prospective, consecutive collection of patient data from the Heart Center Bonn. We identified patients with symptomatic TR who underwent TEER interventions from June 2015 to May 2021. All included patients were considered as inoperable or at high-risk for surgery by the interdisciplinary heart team. After a standardized diagnostic workup, including transthoracic (TTE) echocardiography, left- and right-heart catheterization, the patient’s anatomical suitability for a TEER system was assessed using CT images and transesophageal echocardiography (TEE).

The indication for a tricuspid valve intervention was evaluated based on the current guidelines, and the decision to perform transcatheter tricuspid valve interventions as well as the device selection was made by the interdisciplinary heart team. The registry was approved by the local ethics committee. This study was conducted in accordance with the Declaration of Helsinki and its amendment, and all patients provided written informed consent.

### Procedure

The TEER procedure was performed with either the MitraClip System (Abbott Structural Heart, Santa Clara, CA, USA), TriClip System (Abbott Structural Heart, Santa Clara, CA, USA) or PASCAL Implant System (Edwards Lifesciences, Irvine, CA, USA). The details of each device system and procedure have previously been well described [3, 21–24].

### Echocardiographic assessments

Comprehensive TTE and TEE were performed at baseline, and at discharge and 1-year follow-up a comprehensive TTE was performed according to the current guidelines. The severity of TR was graded by the current recommendations using the five-scaled grading scheme as described in detail before [25]. All measurements were reviewed by two independent cardiologists dedicated to echocardiographic evaluation.

### BMI—measurements and definitions

Underweight was defined as BMI < 20 kg/m^2^ based on the Academic Research Consortium and 2 [26] and 3 [27] definition of frailty. Four groups were defined, after calculating the preprocedural BMI for each patient: underweight (BMI < 20 kg/m^2^), normal weight (BMI 20 to < 25 kg/m^2^, reference group), overweight (BMI 25 to < 30 kg/m^2^) and obesity (BMI ≥ 30 kg/m^2^).

### Endpoints

The primary endpoint of this study was all-cause mortality within 1 year after the procedure. Secondary outcomes were the reduction of TR, New York Heart Association class (NYHA), major adverse cardiovascular and cerebrovascular events (MACCE) (myocardial infarction and stroke) and changes in right ventricular function, measured by TAPSE and right ventricular fractional area change (RVFAC) at 1-year follow-up.

All patients were followed through interviews at scheduled hospital visits, telephone, or documentation from the referring general practitioners.

### Statistics

#### Statistical analysis

Continuous variables are expressed as the mean ± standard deviation or the median (with a 25–75% interquartile range) depending on the distribution of the variables. Categorical variables are presented as numbers and percentages. Continuous variables were compared with a Student’s *t* test or Mann–Whitney *U* test, while the Chi-squared or Fisher exact tests were used for categorical variables. Cox proportional regression model was conducted to calculate hazard ratios (HR) and 95% confidence intervals (CI) for the primary endpoint for each variable. We entered variables with a *p* value < 0.05 upon univariate analysis into the multivariate model. Survival curves for 1 year after TEER procedure are depicted using Kaplan–Meier method. Two-tailed *p* values < 0.05 were considered statistically significant. All statistical analyses were performed using SSPS statistics version 27.0.

## Results

### Study population

All patients (*n* = 211) who underwent TEER were stratified according to their baseline BMI into four groups: underweight (*n* = 29), normal weight (*n* = 71), overweight (*n* = 64) and obesity (*n* = 47). Overall, the mean age was 78.3 ± 7.2, 55.5% of patients were female and the median EuroSCORE II was 9.6 ± 6.7% and the median TRI-SCORE was 5.2 ± 2.0. 182 (86.3%) of subjects were classified as NYHA functional class III and more. The prevalence of hyperlipidemia (*p* < 0.001) and diabetes (*p* = 0.01) was significantly higher in obese patients. All other comorbidities were without significant differences and in addition there were no statistically significant differences in echocardiographic and laboratory assessment between the four groups at baseline (Table 1).

**Table 1:** Baseline patient characteristics

	All	Underweight	Normal weight	Overweight	Obesity	*p* value
	*n* = 211	*n* = 29	*n* = 71	*n* = 64	*n* = 47	
Age, years	78.3 ± 7.2	78.3 ± 6.32	79.5 ± 7.5	78.1 ± 6.8	76.6 ± 7.4	0.18
Female, *n* (%)	117 (55.5)	19 (65.6)	39 (54.9)	34 (53.1)	25 (53.2)	0.71
BMI, kg/m^2^	26.1 ± 5.5	18.7 ± 1.0	22.9 ± 1.4	27.2 ± 1.3	34.1 ± 3.9	** < 0.001**
Risk stratification						
TRI-SCORE	5.2 ± 2.0	5.8 ± 1.8	5.0 ± 2.1	5.1 ± 2.0	5.5 ± 1.8	0.63
EuroSCORE II, %	9.6 ± 6.7	10.6 ± 7.8	10.4 ± 7.0	8.9 ± 6.5	8.9 ± 5.8	0.39
Comorbidities						
Hypertension, * n* (%)	175 (82.9)	23 (79.3)	56 (78.9)	53 (82.8)	43 (91.4)	0.28
Hyperlipidemia, * n* (%)	110 (52.1)	17 (58.6)	29 (40.8)	28 (43.8)	36 (76.6)	** < 0.001**
Diabetes mellitus, * n* (%)	51 (24.2)	5 (17.2)	10 (14.1)	17 (26.6)	19 (40.4)	**0.01**
Peripheral artery disease, * n* (%)	81 (17.1)	13 (44.8)	27 (38.0)	21 (32.8)	2042.6)	0.63
CAD, * n* (%)	117 (55.5)	15 (51.7)	39 (54.5)	32 (50.0)	31 (66.0)	0.39
Prior MI, * n* (%)	57 (27.0)	9 (31.0)	19 (26.8)	16 (25.0)	13 (27.7)	0.95
COPD, * n* (%)	39 (18.5)	7 (24.1)	8 (11.3)	14 (21.9)	10 (21.3)	0.26
NYHA ≥ III, * n* (%)	182 (86.3)	28 (15.4)	58 (31.9)	57 (31.3)	39 (21.4)	0.19
Atrial fibrillation, * n* (%)	195 (92.4)	26 (89.7)	64 (90.1)	61 (95.3)	44 (93.6)	0.70
CIED, * n* (%)	71 (33.6)	10 (34.5)	19 (26.8)	23 (35.9)	19 (40.4)	0.46
Smoking, * n* (%)	9 (4.3)	2 (6.9)	3 (4.2)	3 (4.7)	1 (2.1)	0.74
Smoking history^a^, * n* (%)	47 (22.3)	9 (31.0)	15 (21.1)	12 (18.8)	11 (23.4)	0.61
Prior surgery/interventions						
Prior cardiac surgery, * n* (%)	128 (59.2)	17 (58.6)	45 (63.3)	35 (54.7)	28 (59.6)	0.77
Prior TAVI, * n* (%)	13 (6.2)	1 (3.4)	9 (12.7)	2 (3.1)	1 (2.1)	0.08
Previous MV intervention, * n* (%)						0.13
None	148 (70.4)	25 (86.2)	48 (67.6)	40 (62.5)	35 (74.5)	
TEER	61 (28.9)	4 (13.8)	23 (32.4)	22 (34.4)	12 (25.5)	
Replacement	1 (0.5)	0	1 (1.4)	0	0	
Other	1 (0.5)	1 (3.4)	0	0	0	
Heart failure medication, * n* (%)						
Beta-blockers	200 (94.8)	26 (89.7)	68 (95.8)	61(95.3)	45 (95.7)	0.80
ACEi/ARB	209 (99.1)	28 (96.6)	71 (100.0)	63 (98.4)	47 (100.0)	0.86
Loop diuretic	200 (94.8)	25 (86.2)	68 (95.8)	61 (95.3)	46 (97.8)	0.15
MRA	191 (90.5)	26 (89.7)	65 (91.5)	59 (92.2)	41 (87.2)	
SGLT-2 inhibitor	172 (81.5)	23 (79.3)	58 (81.7)	51 (79.7)	40 (85.1)	
Laboratory parameters						
Creatinine, mg/dl	1.5 ± 0.9	1.4 ± 0.6	1.4 ± 0.7	1.6 ± 0.9	1.8 ± 1.1	0.11
eGFR, ml/min/m^2^	50.0 ± 23.9	55.4 ± 25.8	53.8 ± 25.7	48.8 ± 21.8	43.6 ± 21.7	0.10
NT-proBNP, pg/ml	4296 [3094, 5498]	3526 [1568, 5484]	5282 [2716, 7847]	3082 [2232, 3931]	4779 [1494, 8065]	0. 48
Bilirubin, mg/dl	0.88 ± 0.6	1.0 ± 0.6	0.9 ± 0.6	0.8 ± 0.6	0.8 ± 0.6	0.39
Echocardiographic findings						
LVEF, %	54.4 ± 10.3	53.2 ± 11.2	54.4 ± 11.2	53.8 ± 10.5	55.9 ± 7.8	0.66
RVFAC, %	43.2 ± 9.6	42.7 ± 11.9	43.1 ± 9.7	43.1 ± 9.3	43.1 ± 9.3	1.00
TAPSE, mm	17.9 ± 5.1	17.2 ± 5.9	18.9 ± 5.4	18.0 ± 4.3	16.8 ± 5.0	0.16
TR severity, * n* (%)						0.48
Severe	96 (45.5)	13 (13.7)	36 (50.7)	26 (40.6)	21 (44.7)	
Massive	69 (32.7)	8 (40.8)	24 (33.8)	19 (29.7)	18 (38.3)	
Torrential	46 (21.8)	8 (27.6)	11 (15.5)	19 (29.7)	8 (17.0)	
SPAP, mmHg	36.8 ± 14.2	34.3 ± 9.7	37.3 ± 15.6	35.4 ± 13.9	39.4 ± 14.7	0.51

Values are either *n* (%), mean ± SD, or median [interquartile range]

*BMI* body mass index, *EuroSCORE* European System for Cardiac Operative Risk Evaluation, *CAD* coronary artery disease, *MI* myocardial infarction, *COPD* chronic obstructive pulmonary disease, *NYHA* New York Heart Association, *CIED* cardiac implantable electronic device, *TAVI* transcatheter aortic valve implantation, *MV* mitral valve, *TEER* transcatheter edge-to-edge repair, *ACEi/ARB* angiotensin-converting enzyme inhibitor/angiotensin receptor blocker, *MRA* mineralcorticoid receptor antagonist, *SGLT* Sodium-glucose cotransporter 2, *eGFR* estimated glomerular filtration rate, *NT-proBNP* N-terminal pro-B-type natriuretic peptide, *LVEF* left-ventricular ejection fraction, *RVFAC* right ventricular fractional area change, *TAPSE* tricuspid annular plane systolic excursion, *TR* tricuspid regurgitation, *SPAP* systolic pulmonary artery pressure

^a^Smoking history: defined as a smoking cessation of ≥ 5 years

### Periprocedural findings

Periprocedural findings are summarized in Table 2. Most cases of TEER were treated with the TriClip system (51.2%, Abbott Structural, Santa Clara, CA, USA), followed by the PASCAL Implant System (25.1%, Edwards Lifesciences, Irvine, CA, USA) and the MitraClip system (23.7%, Abbott Structural, Santa Clara, CA, USA). The mean number of implanted devices and implantation failure was comparable between the groups with 1.8 ± 0.8 devices per procedure (*p* = 0.06 and *p* = 0.07). Extensive bleeding events occurred in two patients (one patient with normal weight and one patient with overweight) without fatal outcome. These patients required blood transfusion. There was a tendency towards more failed intervention in the obese group, mainly driven through a lesser postprocedural reduction of TR grade (definition of failed procedure: reduction of TR grade < 2 grades or no device implantation)—without statistical significance (*p* = 0.06).

**Table 2:** Outcome parameters

	All	Underweight	Normal weight	Overweight	Obesity	*p* value
	*n* = 211	*n* = 29	*n* = 71	*n* = 64	*n* = 47	
Procedural findings						
Failed intervention, *n* (%)	17 (8.1)	2 (6.9)	5 (7.0)	2 (3.1)	8 (17.0)	0.06
Implanted devices	1.8 ± 0.8	1.7 ± 0.9	1.8 ± 0.8	2.0 ± 0.8	1.6 ± 0.9	0.07
Procedure time, min	50.6 ± 25.8	47.8 ± 24.3	47.1 ± 21.7	51.9 ± 25.4	56.3 ± 32.3	0.28
Bleedings, *n* (%)						0.12
Minor	8 (3.8)	2 (6.9)	1 (1.4)	4 (6.2)	1 (2.1)	
Major	8 (3.8)	3 (10.3)	2 (2.8)	3 (4.7)	0	
Extensive	2 (0.9)	0	1 (1.4)	1 (1.6)	0	
Myocardial infarction, *n* (%)	0	0	0	0	0	1.0
Stroke, *n* (%)	0	0	0	0	0	1.0
1-Year mortality						
All-cause mortality, *n* (%)	39 (18.5)	11 (37.9)	8 (11.3)	6 (9.3)	14 (29.8)	** < 0.001**
Cardiovascular death, *n* (%)	19 (48.7)	7 (24.1)	5 (7.0)	4 (6.3)	3 (6.4)	** < 0.01**
Non-cardiovascular death, *n* (%)						
Unknown	10	3	1	1	5	
Sepsis/MODS	6		2		4	
Gastrointestinal bleeding	2				2	
Pneumonia	1			1		
Malignancy	1	1				

	All	Underweight	Normal weight	Overweight	Obesity	*p* value
	*n* = 172	*n* = 18	*n* = 63	*n* = 58	*n* = 33	
Outcome at 1-year follow-up						
NYHA class, *n* (%)						0.86
I	68 (68.4)	8 (44.4)	27 (44.3)	19 (32.8)	14 (29.8)	
II	99 (57.6)	10 (55.5)	33 (52.4)	37 (63.8)	19 (40.4)	
III	5	0	3	2	0	
Echocardiographic findings						
TR severity, *n* (%)						0.67
Mild	67 (40.0)	5 (27.8)	27 (42.9)	22 (37.9)	13 (39.4)	
Moderate	84 (48.8)	11 (61.1)	27 (42.9)	30 (51.7)	16 (34.0)	
Severe	19 (9.0)	1 (5.6)	8 (12.7)	6 (10.3)	4 (12.1)	
Massive	1 (0.6)	1 (5.6)	0	0		
Torrential	0	0	0	0	0	
RVFAC, %	47.6 ± 6.9	47.7 ± 7.2	46.9 ± 6.8	47.2 ± 6.9	49.6 ± 6.9	0.33
TAPSE, mm	18.2 ± 3.3	16.9 ± 3.2	18.6 ± 3.2	18.2 ± 3.2	17.9 ± 3.7	0.36
SPAP, mmHg	36.0 ± 15.6	28.9 ± 11.3	36.0 ± 15.6	35.1 ± 12.0	42.2 ± 22.9	0.29

Values are either *n* (%), mean ± SD, or median [interquartile range]

*MODS* multiple organ dysfunction syndrome, *NYHA* New York Heart Association, *TR* tricuspid regurgitation, *SPAP* systolic pulmonary artery pressure, *RVFAC* right ventricular fractional area change, *TAPSE* tricuspid annular plane systolic excursion

### TR reduction

**Fig. 1:**
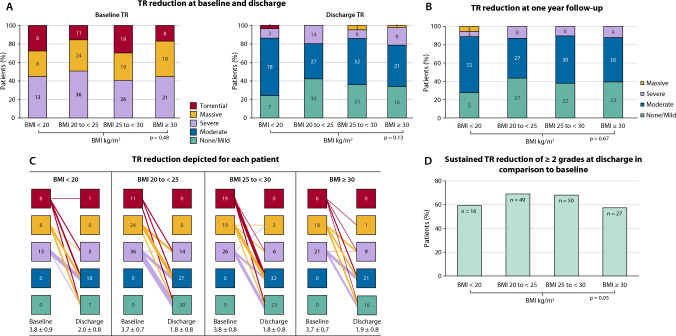
Tricuspid regurgitation (TR) reduction after transcatheter edge-to-edge repair (TEER) stratified according to body mass index (BMI) classes at discharge **a** TR reduction at baseline and discharge, **b** TR reduction depicted for each patient, **c** TR reduction at one year follow-up, **d** sustained TR reduction of ≥ 2 grades at discharge in comparison to baseline

All patients had ≥ severe TR at baseline according to the five-scale grading scheme: 21.8% were graded torrential, 32.7% massive and 45.5% severe. There were no significant differences between the stratified BMI subgroups (*p* = 0.48). At discharge, TR severity was significantly reduced in all subgroups (Fig. 1a, b). In addition, there were no differences between the groups in TR severity at discharge and at 1 year-follow-up (*p* = 0.13 and *p* = 0.67, respectively) (Fig. 1c). 143 (67.8%) of subjects had a sustained TR reduction of ≥ 2 grades at discharge in comparison to baseline. Stratified according to the four BMI classes, underweight and obese patients showed significant lower rates of TR reduction ≥ 2 grades at discharge: 55.2% vs. 70.4% vs. 78.1%. vs. 57.4%; *p* = 0.05 (Fig. 1d).

### NYHA functional class

**Fig. 2:**
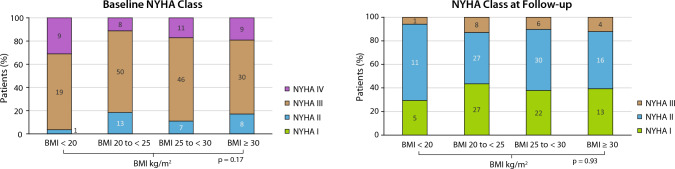
NYHA classes at baseline and one year follow-up stratified according to body mass index (BMI)

Stable and significant improvements in NYHA functional class were found for all patients without significant differences among the subgroups at baseline (*p* = 0.17). At 12 months, 167 of 172 patients (97.1%) were in NYHA functional classes I or II, similar according to BMI subgroups (*p* = 0.93) (Fig. 2).

### Clinical outcomes

**Fig. 3:**
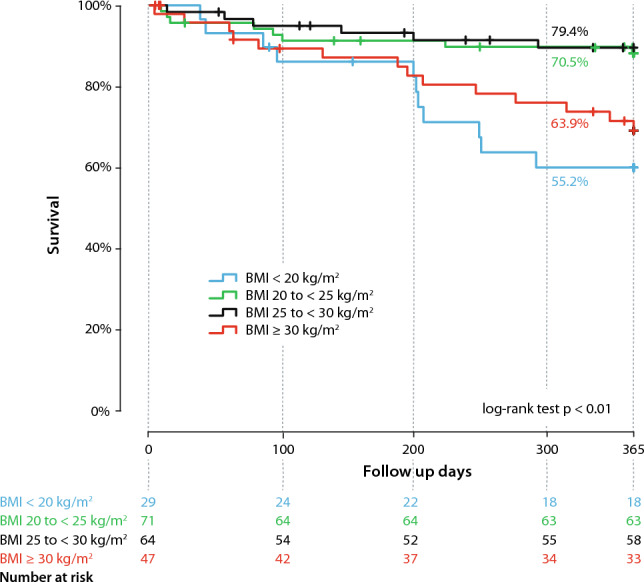
Kaplan–Meier curves for the endpoint of mortality in patients with tricuspid regurgitation (TR) after transcatheter edge-to-edge repair (TEER) stratified by body mass index (BMI)

In the overall cohort, the primary endpoint (all-cause mortality at 1-year follow-up) was observed in 18.5% (39/211) of the patients. Kaplan–Meier curves showed that underweight and obese patients appeared to have a significantly higher all-cause mortality within 1 year after the procedure, compared to normal and overweight patients (*p* log rank < 0.001), 37.9% of the underweight group and 29.8% of the obese group died within 1 year (Fig. 3).

**Fig. 4:**
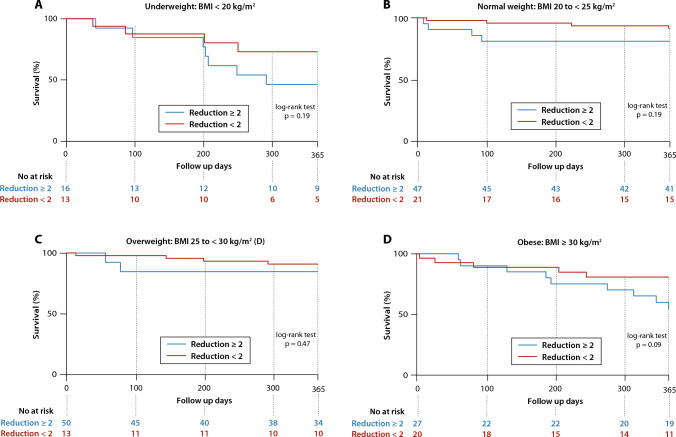
Kaplan–Meier curves for the endpoint of mortality in patients with tricuspid regurgitation (TR) after transcatheter edge-to-edge repair (TEER) stratified by body mass index (BMI) according to TR reduction of of ≥ 2 grades at discharge **a** underweight: BMI < 20 kg/m^2^, **b** normal weight: BMI 20 to < 25 kg/m^2^, **c** overweight: BMI 25 to < 30 kg/m^2^, **d** obese: BMI ≥ 30 kg/m^2^

Patients with a TR reduction of ≤ 2 grades showed a tendency towards a higher 1-year mortality stratified by BMI groups (Fig. 4).

In a multivariable cox proportional hazard model, underweight patients (3.88, 95% CI 1.64–7.66; *p* < 0.01) and obese patients (HR 3.24, 95% CI 1.37–9.16; *p* < 0.01) significantly revealed to be associated with an increased risk of reaching the primary endpoint. COPD (HR 2.52, 95% CI 1.14–5.56; *p* = 0.02) and a depressed left-ventricular ejection fraction (HR 3.28, 95% CI 1.21–8.86; *p* = 0.02) were also identified as predicting factors for reaching the primary endpoint in our cohort (Table 3).

**Table 3:** Cox regression of prediction for 1-year mortality

	Univariable			Multivariable		
	HR	95% CI	*p* value	HR	95% CI	*p* value
Demographic parameters						
Age	1.00	0.98–1.04	0.01	1.06	0.99–1.13	0.08
Female sex	0.72	0.38–1.34	0.31			
Coronary artery disease	1.39	0.73–2.66	0.31			
COPD	2.80	1.44–5.44	**0.002**	2.52	1.14–5.56	**0.02**
CIED	1.60	0.85–3.01	0.16			
Hyperlipidemia	1.16	0.61–2.18	0.66			
Diabetes	1.33	0.66–2.67	0.42			
Echocardiographic parameters						
LVEF < 40%	2.46	1.08–5.57	**0.02**	3.28	1.21–8.86	**0.02**
TAPSE, over 15 mm	1.47	0.25–0.91	**0.02**			
RVFAC_path	1.72	0.85–3.49	0.14			
TR, torrential	1.01	0.47–2.0	0.98			
BMI						
BMI under 20 kg/m^2^	2.66	1.32–5.35	**0.006**	3.88	1.64–7.66	** < 0.01**
BMI over 30 kg/m^2^	1.98	1.03–3.81	**0.01**	3.24	1.37–9.16	** < 0.01**

Significant parameters are depicted bold

*COPD* chronic obstructive pulmonary disease, *LVEF* left-ventricular ejection fraction, *TAPSE* tricuspid annular plane systolic excursion, *RVFAC* right ventricular fractional area change, *TR* tricuspid regurgitation, *BMI* body mass index

## Discussion

Up to now, little is known regarding the influence of BMI on procedural outcome after TEER in patients with symptomatic TR. This study reports the implications of different BMI categories on the outcome of TEER procedures in patients with TR which to the best of our knowledge has not been published before. Our findings can be summarized as follows:

1. The prevalence of underweight in patients with symptomatic TR receiving TEER treatment was 13.7% (*n* = 29), additionally, prevalence of obese patients measured 22.3% (*n* = 47).
2. Except for a significant higher prevalence of hyperlipidemia and diabetes mellitus type 2 in the obese subcohort, baseline characteristics were comparable throughout the four BMI subcohorts.
3. For patients with symptomatic TR undergoing TEER, underweight (BMI < 20 kg/m^2^) and obesity (BMI > 30 kg/m^2^) were associated with significantly increased risk of 1-year all-cause mortality.
4. Regarding postprocedural complications, no differences in vascular and bleeding complications were documented.
5. Underweight and obese patients showed inferior rates of TR reduction of ≥ 2 grades after TEER.

BMI is a comparatively easy-to-asses parameter and routinely documented during hospital admission. Both underweight and overweight have previously been reported to be strong predictors for mortality in cardiovascular disease [14]. In our analysis, the absolute number of underweight patients was 29 (13.7%) and 47 (22.3%) of patients were obese. There were no significant differences regarding age, gender and surgical risk scores at baseline, however, concerning relevant co-morbidities, hyperlipidemia and diabetes mellitus were more frequent in obese patients compared the other subcohorts (Table 1). The prevalence of underweight and obesity and a potential influence on outcome have not been reported in previously published trials examining patients with TR undergoing TEER or other interventional reconstruction systems in the treatment of TR, for example in TRILUMINATE [3, 22], TRI-Repair [28], and studies focusing on TEER with PASCAL Implant System [29] or the MitraClip System [30] systems BMI values were not reported, respectively. Aurich et al. [31] reported BMI values in a small cohort of 16 patients with ≥ severe TR who underwent TEER with the PASCAL Ace Implant System: the mean BMI was 26 ± 3 kg/m^2^ in the complete cohort, there were no differences between the failed (*n* = 5) and successful (*n* = 11) intervention groups (BMI 24 ± 3 kg/m^2^ vs. BMI 26 ± 4 kg/m^2^; *p* = 0.24).

### Obesity

Obesity has become an increasingly common chronic condition in the western civilization which is associated with significant morbidity and mortality [32–34]. It has also been characterized as a major and also modifiable risk factor for cardiovascular morbidity and mortality by the American Heart Association/American College of Cardiology and the Nutrition Council of the American Heart Association [35]. However, a considerable number of studies reported a beneficial effect of overweight and obesity on survival in patients with established heart disease in general (e.g. CAD, atrial fibrillation) and specifically in patients undergoing interventional procedures [36–39], such as transcatheter aortic valve replacement [40], TEER for symptomatic mitral regurgitation [17] and in patients with heart failure [41, 42]—in these studies, obesity was characterized inconsistently but mainly as a BMI ≥ 30 kg/m^2^. The phenomenon of a beneficial effect of overweight and obesity on survival was termed the *obesity paradox*, and its validity is still under discussion in the literature.

In our analysis, obesity—defined as a BMI ≥ 30 kg/m^2^—was associated with inferior survival (HR 3.24, 95% CI 1.37–9.16; *p* < 0.01) compared to normal weight and overweight patients. One reason in this regard might trace back to the fact that the underlying pathology mainly consists of a leading right heart disease and right heart failure whereas the studies showing a beneficial effect of obesity examined leading left heart diseases and valvulopathies, respectively. Thus, a different effect of overweight on outcome in patients with right heart failure and TR does not categorically contradict the proposed *obesity paradox* as it merely stresses the different disease entities of right and left sided valvulopathies and heart failure and their different clinical presentation and pathophysiology, respectively.

The investigators against the validity of the *obesity paradox* in the aforementioned cohorts argue that the obese population is younger, seeks medical care earlier, is treated medically more aggressively, and therefore, benefits more from medical and interventional treatment [36], thus obesity being merely a surrogate. Indeed, in our cohort obese patients were slightly but not statistically significant younger (mean age in: obesity 76.6 ± 7.4 years vs. overweight 78.1 ± 6.8 years vs. normal weight 79.5 ± 7.5 years vs. underweight age 78.3 ± 6.3 years; *p* = 0.18), but did not show a beneficial outcome. A possible explanation could be inferior rates of pronounced TR reduction (≥ 2 TR grades to baseline) of 42.6% in patients with the highest BMI. Additionally, rates of failed procedures (no device implantation or TR reduction ≤ 2 grades to baseline) appeared to be slightly higher in obese patients (17%, 8/47)—mainly driven by fewer cases with a pronounced TR reduction postprocedurally compared to baseline, but without statistical significance (*p* = 0.06).

A possible explanation might also be a potential difference in leaflet characteristics in obese patients compared to normal- and overweight patients making grasping more difficult, but by now this cannot easily be examined. Moreover, there were no significant differences regarding occurrence of SLDA (single leaflet device attachment) or number of implanted devices.

Additionally, we hypothesize that increasing BMI may have associated independent confounders not demonstrated in our study population for which it was potentially not powered enough and will be addressed in follow-up studies in further detail—such as leaflet characteristics, difficulty with catheter—or device guiding and navigation, challenging imaging, or different atrial and ventricular volumetries and TV annulus anatomies and dimensions.

Lastly, our analysis documented an increased 1-year all-cause mortality of underweight and obese patients receiving TEER for significant TR. Obese patients showed a comparable cardiovascular mortality after 1 year compared to the normal and overweight cohorts. As a main reason we postulate a potential underestimation of cardiovascular mortality in the obese cohort as in 5 deaths (10.6%) reasons for death remained unknown (in comparison to 4.7%, 10.3%, 1.4% and 1.5% for all, underweight, normal- and overweight patients).

### High underweight mortality

Underweight has been previously documented as a risk factor for cardiovascular mortality [14]. However, there is no generally accepted definition of underweight—especially in the elderly, thus limiting comparability throughout the studies. Kalbacher et al. [17] reported underweight patients with mitral regurgitation undergoing TEER procedure with the MitraClip system to be exposed to increased short- and long-term mortality. In our cohort, underweight was associated with inferior 1-year survival (HR 3.88; 95% CI 1.64–7.66; *p* < 0.01) compared to normal weight and overweight patients. A significantly higher rate of cardiovascular death was documented in the underweight cohort compared to the other groups (24.1% vs. 7.0% vs. 6.3% vs. 6.4%; *p* < 0.01). Overall, only limited data exist about potential reasons as to why underweight is associated with increased mortality rates in cardiovascular patients. It cannot be excluded that underweight might act as a surrogate parameter for frailty as the most common definition used for frailty includes underweight and weight-loss, respectively—as well as serum-albumin levels, grip strengths, cognitive function and mobility (6 min-walking test) [26]. Moreover, underweight might effect a decreased capacity for reconvalescence and an overall reduced physiological reserve which might partly be an explanation for an increased short-term mortality. In addition, increased vulnerability to stressors has been discussed.

## Limitations

Several limitations have to be taken into account. The modest sample size, the retrospective and single-center character reduce the generalizability and thus, this study should be considered rather hypothesis generating underlining the need for validation in a prospective and multi-center trial design.

## Conclusions

In patients undergoing TEER for significant symptomatic TR, underweight (BMI < 20 kg/m^2^) and obesity (BMI > 30 kg/m^2^) were associated with significantly higher 1-year all-cause mortality compared to normal weight and overweight patients with no striking differences in baseline characteristics. There were no differences in bleeding and vascular complications. Changes in NYHA Functional Class and over-all TR reduction were comparable across all subgroups.

Moreover, after interventional treatment for significant TR, lower rates of pronounced TR reduction (≥ 2 TR grades reduction to baseline) were observed in underweight and obese patients compared to normal weight (BMI 20 to < 25 kg/m^2^) patients.

Considering the well‐documented association between obesity and cardiovascular morbidity and mortality [34, 43, 44] and the expanding nature of obesity as an endemic healthcare problem, it is reasonable to expect an increasing number of obese patients with severe and symptomatic TR being referred for TEER. Thorough preinterventional evaluation should be considered to identify those patients who would benefit most from interventional TR treatment. Identification and incorporation of potential risk factors such as obesity and underweight, frailty and malnutrition, respectively, should be included during patient evaluation and might contribute regarding optimal patient selection. However, there is a great need for detailed multi-center and prospective trials to validate the impact of potential risk factors.

### Supplementary Information

Below is the link to the electronic supplementary material.

Supplementary file1 (DOCX 14 KB)

## Data Availability

The dataset supporting the conclusions of this article are included within the article. The datasets during and/or analyzed during the current study are available from the corresponding author on reasonable request.

## Abbreviations

LVEF: Left-ventricular ejection fraction
LV: Left ventricle
MR: Mitral regurgitation
NYHA: New York Heart Association
RVFAC: Right-ventricular fractional area change
TAPSE: Tricuspid annular plane systolic excursion
TEER: Transcatheter edge-to-edge repair
TMVR: Transcatheter mitral valve replacement
TEE: Transesophageal echocardiography
TR: Tricuspid regurgitation
TTE: Transthoracic echocardiography
BMI: Body mass index
