# Supplementary material for: Impact of body mass index in patients with tricuspid regurgitation after transcatheter edge-to-edge repair
Source: Clin Res Cardiol. 2023 Oct 4;113(1):156–67. doi: 10.1007/s00392-023-02312-2 (PMC10808352; doi:10.1007/s00392-023-02312-2)
Supplement: Supplementary file 1 — Supplementary file1 (DOCX 14 KB) [file 392_2023_2312_MOESM1_ESM.docx]

**Supplemental Table 1. 30-day outcome parameters**

| **30-day mortality, n (%)** | **6/211 (2.8)** | **-** | **3/71 (4.2)** | **1/64 (1.6)** | **2/47 (4.3)** | **0.61** |
| --- | --- | --- | --- | --- | --- | --- |
|  | All | Underweight | Normal weight | Overweight | Obesity |  |
|  | n = 205 | n = 29 | n = 68 | n = 63 | n = 45 | p value |
| Myocardial infarction, n (%) | - | - | - | - | - | 1.0 |
| Stroke, n (%) | - | - | - | - | - | 1.0 |
| NYHA class, n (%) |  |  |  |  |  | 0.61 |
| I | 107 (52.2) | 10 (34.5) | 31 (45.6) | 25 (39.7) | 20 (44.4) |  |
| II | 95 (46.3) | 15 (51.7) | 22(32.4) | 28 (44.4) | 21 (46.7) |  |
| III | 3 (1.5) | 4 (13.8) | 7 (10.3) | 5 (7.9) | 4 (8.9) |  |
| ***Echocardiographic findings*** |  |  |  |  |  |  |
| TR severity, n (%) |  |  |  |  |  | 0.43 |
| mild | 90 (43.9) | 12 (41.4) | 27 (39.7) | 27 (42.9) | 20 (44.4) |  |
| moderate | 110 (53.7) | 17 (58.6) | 33 (48.5) | 32 (50.8) | 18 (40.0) |  |
| Severe | 4 (2.0) | - | 8 (11.8) | 4 (6.3) | 7 (15.6) |  |
| Massive | 1 (0.5) | - | - | - | - |  |
| torrential | - | - | - | - | - |  |
| RVFAC, % | 48.6 ± 6.7 | 48.7 ± 7.4 | 47.9 ± 6.7 | 47.9 ± 7.1 | 48.7 ± 6.9 | 0.43 |
| TAPSE, mm | 19.2 ± 3.4 | 15.9 ± 2.2 | 18.4 ± 3.0 | 18.5 ± 3.3 | 18.0 ± 3.6 | 0.40 |
| SPAP, mmHg | 35.0 ± 14.6 | 29.9 ± 12.4 | 37.1 ± 16.6 | 34.1 ± 14.1 | 40.2 ± 17.3 | 0.21 |
| Values are either n (%), mean ± SD, or median [interquartile range].  Legends: NYHA = New York Heart Association; TR = tricuspid regurgitation; SPAP = systolic pulmonary artery pressure; RVFAC = right ventricular fractional area change; TAPSE = tricuspid annular plane systolic excursion. | | | | | | |
